# Growth Impairment and Nutritional Deficiencies in a Cow's Milk-Allergic Infant Fed by Unmodified Donkey's Milk

**DOI:** 10.1155/2011/103825

**Published:** 2011-09-06

**Authors:** Enza D'Auria, Marzia Mandelli, Patrizia Ballista, Francesco Di Dio, Marcello Giovannini

**Affiliations:** Unit of Allergy, Department of Pediatrics, San Paolo Hospital, The University of Milan, 20122 Milan, Italy

## Abstract

We report a case of growth impairment and nutritional deficiencies in a five-month infant fed by unmodified donkey's milk. We discuss the energy and macronutrient daily intake from donkey's milk and the nutritional consequences that can occur if this kind of milk is used unmodified in the first year of life.

## 1. Introduction

Cow's milk allergy (CMA) is frequently observed during the first year of life when nutritional requirements are critical [1]. Successful therapy depends on completely eliminating cow's milk proteins from the child's diet. 

In those cases where breastfeeding is not available, the replacement food should be hypo- or anallergenic, non-cross-reactive with cow's milk, nutritionally adequate, and palatable, the latter being fundamental in view of the young age of these patients. 

Extensively hydrolysed formulas (eHFs) are recommended as first choice for CMA treatment by the European Society for Paediatric Allergology and Clinical Immunology (ESPACI), the European Society for Paediatric Gastroenterology, Hepatology and Nutrition (ESPGHAN) [2], and the American Academy of Pediatrics (AAP) [3]; however, they are not tolerated by all children suffering from CMA [4].

Only amino-acid formulas can be considered nonallergenic. They can be employed in children intolerant to extensively hydrolysed formulas or as first-line therapy in more severe CMA cases complicated with malabsorption and poor growth [5]. 

Extensively hydrolysed formulas and amino-acid-based formulas have been demonstrated adequate to promote normal growth [3, 6, 7]. 

However, these formulas both have some drawbacks like unpleasant taste and high costs.

Soy-based-formula (SF) should not be used in infants with food allergy during the first six months of life [8]; SFs are also contraindicated for the treatment of children with some forms of non-IgE-associated gastrointestinal CMA [3]. 

Moreover, controversy has developed over the adequacy and safety of SF. Most concerns refer to the high concentration of both phytoestrogens, the long-term effects of which are unknown, and phytate that may interfere with iodine metabolism [8].

Hydrolysed rice protein formulas have become available and have been shown to be well tolerated by infants affected from CMA in prospective randomized clinical studies [9, 10]. Children receiving this formula showed similar growth to those receiving an eHF [11, 12]. However, few data are present in literature regarding this type of formula.

For all the above reasons, the possibility of using milk from other mammalian species has been examined.

Goat's milk and sheep's milk are generally contraindicated as their protein have shown extensive cross-reactivity with cow's milk proteins (CMPs) both in vitro and in vivo [13, 14]. Mare's milk is much closer to human milk than to CM and it has been demonstrated to be tolerated by some children with severe IgE-mediated CMP [15]; however, few data exist and its availability is limited. 

Donkey's milk (DM) has a lower protein content than other mammalians' milk, and also its proteomic profile is similar to breast milk [16], it has an acceptable taste, and it is less expensive than eHF. However, it is poor in lipids, and it has a low caloric value with respect to human milk and other mammalians' milk [17], that represents a limit to its employment in the toddler's diet.

With regard to this, we here report a case that describes an example of nutritional imbalance due to the use of unmodified donkey's milk in a 5-month cow's milk-allergic infant.

## 2. Case Report

The patient is an Italian female who first attended our allergy clinic at 5 months of age. She was born at 37 week gestation, weighing 2970 gr to Italian nonconsanguineous parents. 

She was exclusively breastfed until first month of life, and then she was fed with a starting formula (Nidina 1; Nestlè) for fifteen days only. 

At the age of two months, she was first admitted with cyanosis and vomiting. In that occasion a diagnosis of suspicion of gastroesophageal reflux was made. It was not possible to make a definitive diagnosis because the mother refused to submit the child to esophageal pH test. The child was discharged with an antiregurgitation formula (Nidina Comfort; Nestlè) that was stopped after twenty days because of the persistence of symptoms.

An extensively hydrolysed whey formula (Alfarè; Nestlè) was then introduced in the diet; a treatment with omeprazole and a gastroprokinetic agent was also started. 

The mother did not observe any improvement in her symptoms. Therefore, at three months of life she introduced donkey's milk in the infant's diet on her own initiative. She felt donkey's milk would be more nutritionally complete than whey hydrolysed formula, besides being more palatable. Parents ordered donkey's milk to a farm placed in Tuscany on internet. 

Although the frequency of regurgitation and vomiting has decreased without completely resolving, weight showed a progressive impairment. 

At her first attendance at our allergy clinic, the patient appeared thin and wasted. The weight (5300 g) was below the 3rd percentile, although the length (63.5 cm) was on 25th percentile and head circumference (43 cm) was on 50°–90° percentile. Positive findings included pallor, generalized hypotonia, abdominal distension, and decreased muscle bulk.

A dietary history revealed she averagely consumed 600 mL of ass's milk daily and some tablespoons of soft foods such as cereals and vegetables for lunch. Daily caloric intake was estimated to be 276,45 Kcal, which is 42% of the recommended dietary allowance (RDA). The diet was high in proteins: 8,39 g, which is 130% of RDA. Fat intake was 6,75 g. The daily iron intake was also inadequate: 3,08 mg, which is 44% of RDA. Finally, much lower than the recommended dietary allowance was also the intake of calcium: 380 mg, which is 63,3% of RDA (Table 1).

A routine laboratory evaluation revealed microcytic hypochromic anaemia (Hb 8,1 mg/dL, MCV 64,1 fl, MCH 22 pg, serum iron level 25 uU/dL). Low levels of prealbumin (11,7 mg/dL) and some amino acids (alanine, glycine), both markers of caloric malnutrition, were also observed.

Albumin was probably not decreased because it is relatively insensitive to changes in nutrition as a consequence of its long half-life and its relatively large body pool.

It was therefore possible to come to the conclusion that the infant's iron deficiency anaemia was probably caused by the consumption of ass's milk (Table 2) which contains very low quantities of iron for the infant's age.

She performed an open oral food challenge with cow's milk-based starting formula that provoked vomiting at the third dose. For this reason a cow's milk-protein-free diet was prescribed, and she was started with an elemental formula (Neocate; Nutricia).

In the following weeks the growth of the infant improved: after one month, she reached 6030 g (3rd–10th percentile). Later on, her haematological and metabolic plasmatic parameters normalized. 

Therefore, she continued with a cow's milk-protein-free diet with good compliance. In March 2009, during a control at our Unit of Allergy, Department of Pediatrics, a further growth has been noticed (weight = 7,750 Kg—10°–25°pc), while the child was undergoing the weaning with the introduction of beef and veal in her diet without any reaction. 

## 3. Discussion

Generally, cow's milk substitutes should be adequate not only from an allergological point of view but also from a nutritional point of view, especially in the first year of life when nutritional requirements are critical for growth and development. 

Few clinical studies evaluated DM tolerability that seems quite good even if it did not achieve the 90% tolerability value required to define a hypoallergenic formula [18, 19]. 

However, there are no clinical studies with an adequate statistical design to evaluate the nutritional efficiency of DM, at least in the first year of life. 

The only three clinical studies with this primary aim have some important drawbacks. The first one described a series of 9 case reports all with gastrointestinal symptoms [20]; the second one had a retrospective design and a small sample size not adequately powered to detect growth difference [21]; finally the third one had a small sample size, and, moreover, it did not consider standardized indices for weight and length (z-scores) [22]. All of these statistical drawbacks call in question the validity of the results. 

Anyway, interest on growth rates in the first year of life has been raised by the observation that a “restricted” growth in this period could affect health outcomes in adulthood [23].

In spite of the paucity of the data, DM has been traditionally used to feed some allergic infants in many southern Italian regions and its use is actually increasing in other settings as it is more available. 

DM is considered “safe” as it is perceived “natural” and more nutritionally complete than special hypoallergenic formulas approved for infancy. For this reason, parents and sometimes pediatricians also consider unlikely the possibility of nutritional deficiencies caused by the use of cow's milk alternatives in the infant's diet, and therefore serious dietary problems may emerge.

DM has a poor lipid content and a low caloric value in addition to a very low iron content; this can lead to caloric malnutrition because recommended dietary allowances are not reached, also in weaned infants as this case highlights.

Accordingly, DM should not be considered an adequate choice for feeding CMA children, at least in the first year of life, until prospective randomized statistically powered clinical trails will evaluate safety profile of this mammal-derived milk accordingly to a recent review by Muraro et al. [13].

If ass's milk is employed in selected cases, it should be adequately supplemented and both nutritional status and growth should be monitored. 

Moreover, in view of this case, we would like to stress the importance to reconsider the priority we allocate to the daily dietary history of allergic children on cow's milk free diet. Knowledge regarding nutrient composition of food consumed is crucial as the exact quantity of special formulas or other milk substitute assumed for a day. 

Finally, all the above considerations make it mandatory that the diet of a cow's milk-allergic infant should be supervisioned by a nutritionist or a dietitian experienced in food allergy.

## Figures and Tables

**Table 1:** Energy and macronutrient daily intake.

	Ass's milk	Other foods	%RDA
Energy (Kcal)	244.8	113.25	70.37
Proteins (g)	10.32	1.51	149.43
Lipids (g)	2.28	5.23	32%
Carbohydrates (g)	41.28	15.2	88%
Iron (mg)	0.06	3.04	44.28
Calcium (mg)	480	60	90

**Table 2:** Comparison between nutritional values (mean per 100 mL) of human milk and donkey's milk.

	Energy (Kcal/L)	Proteins (g/dL)	Lipids (g/dL)	Carbohydrates (g/dL)	Iron (mg/dL)	Calcium (mg/dL)
Ass's milk	408	1.72	0.38	6.88	0.01	80
Breast milk	690	0.9	3.83	6.81	0.04	28

## References

[1] Sampson HA (2004). Update on food allergy. *Journal of Allergy and Clinical Immunology*.

[2] Høst A, Koletzko B, Dreborg S (1999). Dietary products used in infants for treatment and prevention of food allergy. Joint statement of the european society for paediatric allergology and clinical immunology (ESPACI) committee on hypoallergenic formulas and the european society for paediatric gastroenterology, hepatology and nutrition (ESPGHAN) committee on nutrition. *Archives of Disease in Childhood*.

[3] American Academy of Pediatrics (1999). Committee on nutrition. Hypoallergenic infant formulas. *Pediatrics*.

[4] Høst A, Halken S (2004). Hypoallergenic formulas—When, to whom and how long: after more than 15 years we know the right indication!. *Allergy*.

[5] Hill DJ, Murch SH, Rafferty K, Wallis P, Green CJ (2007). The efficacy of amino acid-based formulas in relieving the symptoms of cow’s milk allergy: a systematic review. *Clinical and Experimental Allergy*.

[6] Niggeman B, Christaine B, Dupont C, Hadji S, Arvola T, Isolauri E (2001). Prospective, controlled, multi-center study on the effect of an aminoacid based formula in infants with cow’s milk allergy/intolerance and atopic dermatitis. *Pediatric Allergy and Immunology*.

[7] Sicherer SH, Noone SA, Koerner CB, Christie L, Burks AW, Sampson HA (2001). Hypoallergenicity and efficacy of an amino acid-based formula in children with cow’s milk and multiple food hypersensitivities. *Journal of Pediatrics*.

[8] ESPGHAN Committee on Nutrition, Agostoni C, Axelsson I (2006). Soy protein infant formulae and follow-on formulae: a commentary by the ESPGHAN committee on nutrition. *Journal of Pediatric Gastroenterology and Nutrition*.

[9] Fiocchi A, Travaini M, D’Auria E, Banderali G, Bernardo L, Riva E (2003). Tolerance to a rice hydrolysate formula in children allergic to cow’s milk and soy. *Clinical and Experimental Allergy*.

[10] Fiocchi A, Restani P, Bernardini R (2006). A hydrolysed rice-based formula is tolerated by children with cow’s milk allergy: a multi-centre study. *Clinical and Experimental Allergy*.

[11] Agostoni C, Fiocchi A, Riva E (2007). Growth of infants with IgE-mediated cow’s milk allergy fed different formulas in the complementary feeding period. *Pediatric Allergy and Immunology*.

[12] Reche M, Pascual C, Fiandor A (2010). The effect of a partially hydolysed fomrula based on rice protein in the treatment of infantf with cow’s milk protein allergy. *Pediatric Allergy and Immunology*.

[13] Muraro MA, Giampietro PG, Galli E (2002). Soy formulas and nonbovine milk. *Annals of Allergy, Asthma and Immunology*.

[14] Pessler F, Nejat M (2004). Anaphylactic reaction to goat’s milk in a cow’s milk-allergic infant. *Pediatric Allergy and Immunology*.

[15] Businco L, Giampietro PG, Lucenti P (2000). Allergenicity of mare’s milk in children with cow’s milk allergy. *Journal of Allergy and Clinical Immunology*.

[16] D’Auria E, Agostoni C, Giovannini M (2005). Proteomic evaluation of milk from different mammalian species as a substitute for breast milk. *Acta Paediatrica*.

[17] Salimei E, Fantuz F, Coppola R, Chiofalo B, Polidori P, Varisco G (2004). Composition and characteristics of ass’s milk. *Animal Research*.

[18] Monti G, Bertino E, Muratore MC (2007). Efficacy of donkey’s milk in treating highly problematic cow’s milk allergic children: an in vivo and in vitro study. *Pediatric Allergy and Immunology*.

[19] Vita D, Passalacqua G, Di Pasquale G (2007). Ass’s milk in children with atopic dermatitis and cow’s milk allergy: crossover comparison with goat’s milk. *Pediatric Allergy and Immunology*.

[20] Iacono G, Carroccio A, Cavataio F, Montalto G, Soresi M, Balsamo V (1992). Use of ass’ milk in multiple food allergy. *Journal of Pediatric Gastroenterology and Nutrition*.

[21] Carroccio A, Cavataio F, Montalto G, D’Amico D, Alabrese L, Iacono G (2000). Intolerance to hydrolysed cow’s milk proteins in infants: clinical characteristics and dietary treatment. *Clinical and Experimental Allergy*.

[22] Tesse R, Paglialunga C, Braccio S, Armenio L (2009). Adequacy and tolerance to ass’s milk in an Italian cohort of children with cow’s milk allergy. *Italian Journal of Pediatrics*.

[23] González-Barranco J, Ríos-Torres JM, Castillo-Martínez L (2003). Effect of malnutrition during the first year of life on adult plasma insulin and glucose tolerance. *Metabolism*.

